# Prognostic significance of classified extramural tumor deposits and extracapsular lymph node invasion in T3–4 colorectal cancer: a retrospective single-center study

**DOI:** 10.1186/s12885-015-1885-6

**Published:** 2015-11-06

**Authors:** Tomoki Yamano, Shuho Semba, Masafumi Noda, Mie Yoshimura, Masayoshi Kobayashi, Michiko Hamanaka, Naohito Beppu, Aya Yano, Kiyoshi Tsukamoto, Nagahide Matsubara, Naohiro Tomita

**Affiliations:** 1 Division of Lower Gastrointestinal Surgery, Department of Surgery, Hyogo College of Medicine, 1-1 Mukogawa-cho, Nishinomiya, Hyogo 663-8501 Japan; 2 Department of Pathology, Kobe Ekisaikai Hospital, Kobe, Hyogo Japan

**Keywords:** Colorectal cancer, Extramural tumor deposit, Extracapsular invasion, Prognosis, Classification

## Abstract

**Background:**

Extramural tumor deposits (TDs) and extracapsular lymph node involvement (ECLNI) are considered to be poor prognostic factors in patients with T3–4, N0–2, M0 colorectal cancer (CRC). Although TDs are known to have multiple origins and pleomorphic features, the prognostic significances of the different type of TDs have not yet been established.

**Methods:**

We performed a retrospective review of 385 consecutive patients with T3–4, N0–2, M0 CRC who received curative resection at our institution between 2006 and 2012. We classified the TDs into two groups: invasive-type TD (iTD), which is characterized by the presence of lymphatic invasion, vascular invasion, perineural invasion, or undefined cancer cell clusters and nodular-type TD (nTD), which is characterized by a smooth or irregular-shaped tumor nodule other than an iTD. ECLNI was defined as invasion of cancer cells into capsular collagen tissues or adipose tissues beyond the capsular collagen. Multivariate analyses were used to assess the prognostic significance of iTD, ND, and ECLNI for relapse-free survival (RFS), disease-specific survival (DSS), and sites of recurrence.

**Results:**

In patients without lymph node (LN) metastasis, the incidences of iTD and nTD were both in the range of 2–3 %. Conversely, in patients with LN metastasis, the incidences of iTD, nTD, and ECLNI were 31, 22, and 34 %, respectively. iTD, nTD, and ECLNI were all significant independent adverse factors for RFS in rectal cancer, and were all associated with pT, pN, and LN ratio. iTD was a significant independent adverse prognostic factor for DSS in rectal cancer, metastasis to the liver in colorectal cancer, and distant LN metastasis in colon cancer. ECLNI was a significant independent prognostic factor for RFS in colon cancer.

**Conclusions:**

Classifying TDs and assessing ECLNI may help establish significant prognostic factors for patients with T3–4, N0–2, M0 CRC.

## Background

Colorectal cancer (CRC) is one of the most common malignancies in the world. In Japan, it is the third and second most common cancer in men and women, respectively [1]. Although most patients are diagnosed in the early stages of the disease (without distant metastasis or locally advanced status), some patients develop local or distant recurrence within 5 years of undergoing curative resection; therefore, accurate assessments of the risk of recurrence are important for improving the prognosis of this disease, and the TNM staging system has been revised accordingly every several years. Of note, although the evaluation of N status is especially critical for TNM staging, the definition and significance of extramural tumor deposits (TDs), which affect the N status, remain controversial. Further, extracapsular lymph node involvement (ECLNI) has not yet been adopted as a factor for staging [2].

The presence of TDs in the resected specimen is considered to be a poor prognostic factor [3–8]. In the 5^th^ edition of the TNM classification manual, TDs were originally classified according to size (larger or smaller than 3 mm), whereas in the 6^th^ edition, they were further classified according to shape (smooth or irregular) [9, 10]. In the 7^th^ and most recent edition, TDs are considered as an N factor (N1c) in cases of T1–2 disease without lymph node (LN) metastasis [11]. TDs in T1–2 disease were defined as a T factor or an N factor in the previous versions of the TNM classification. In the 7^th^ and most recent edition, TDs are considered as an N factor (N1c) in cases of T1–4 disease without lymph node (LN) metastasis [11]. However, the effect of TD status is not mentioned for cases of T1–4 disease with LN metastasis [11]. Therefore, we focused on TDs in T3-4 tumor disease with or without lymph node metastasis.

Although TDs are considered to originate from the lymphovascular/perineural invasion of cancer cells [4, 12], the morphology of TDs is so diverse that their origins are unclear in many cases [12, 13]. The most critical problem is distinguishing TDs from LN metastasis, as the number of LN metastases influences staging [13]. Accordingly (as mentioned above), TD and LN metastasis have been distinguished in the TNM staging systems based on the size or shape of the lesion [9, 10], and several previous studies have assessed the independent prognostic significances of various TD classifications, including irregular or smooth nodules, lymphocyte invasion, vascular invasion, and aggressive patterns [5–8].

Moreover, some previous studies have also reported that ECLNI is an adverse prognostic factor, although the TNM system has not yet incorporated ECLNI status into the staging [14–16]. ECLNI is defined as an invasive cancer aggregate beyond the LN capsule. However, Brabender et al. reported on ECLNI comprising TDs without perineural invasion or vessel involvement, suggesting that it is necessary to distinguish between TDs and ECLNI [16].

Considering the issues mentioned above, we undertook the present study to assess the prognostic significance of TD types and ECLNI status in patients with T3–4, N0–2, M0 CRC. For the purposes of our study, TDs were classified into two types: invasive-type tumor deposits (iTDs), which refer to cancer aggregates with lymphovascular or perineural invasion, or clusters of cancer cells and nodule-type tumor deposits (nTD), which refer to other TDs. In this manner, we have classified TDs according to whether their origins are clear, rather than their sizes and shapes.

## Methods

### Patients

We conducted a pathological and clinical review of 385 consecutive patients with T3 (327 patients) or T4 (58 patients) plus N0 (206 patients), N1 (125 patients), or N2 (54 patients) status who received curative resection for colon (289 patients) or rectal (96 patients) cancer at our hospital between 2006 and 2012. This study was approved by the ethics committee of Hyogo College of Medicine. Written informed consent was obtained from all included patients. We excluded patients with CRC who met any of the following criteria: inflammatory bowel disease, such as ulcerative colitis or Crohn’s disease; familial adenomatous polyposis; a history of any other advanced cancer within the 5 years prior to the diagnosis of CRC; preoperative chemotherapy and/or radiation therapy for CRC. The clinical data collected included the patient demographics, the operation performed, adjuvant chemotherapy, and outpatient follow-up information on recurrence and survival. Relapse-free survival (RFS) was defined as the interval (in months) between the date of surgical removal of the primary tumor and the date at which relapse was confirmed or the date of the last follow-up (for censored patients). Disease-specific survival (DSS) was defined as the interval (in months) between the date of surgical removal of the primary tumor and the date of death from cancer or the date of the last follow-up (for censored patients). As adjuvant treatment after surgery, 70 of the 206 (34 %) patients with stage II CRC, and 131 of 179 (73 %) patients with stage III CRC received 5-fluorouracil-based chemotherapy. Recurrence was assessed every 3–6 months based on physical examinations, blood tests, and computed tomography scans. The mean and median follow-up periods were 46.9 and 46.0 months, respectively.

### Pathological examination

**Fig. 1:**
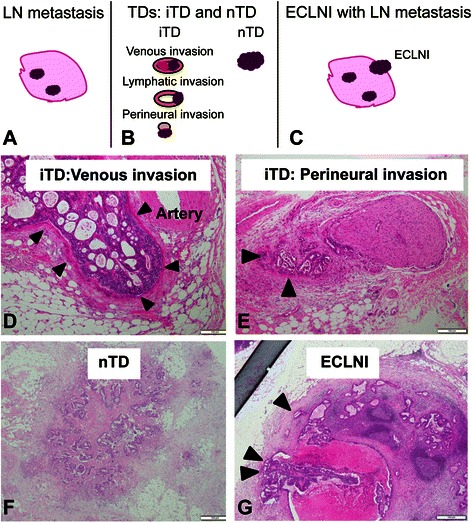
Schematic illustrations and photos. **a** Lymph node (LN) metastasis. **b**, **d**-**f** Extramural tumor deposits (TDs). **c**, **i**, **g** Extracapsular lymph node involvement (ECLNI). TD was classified as invasive type (iTD) (**d**, **e**) or nodular type (nTD) (**f**) in this study

The resected specimens were retrieved and reviewed for routine pathological evaluation by hematoxylin and eosin staining according to the 7^th^ edition of the TNM classification [11]. The specimens that had been harvested for the pathological examination of LN metastasis were further reviewed to evaluate the presence of TDs and ECLNI. As mentioned above, TDs were classified as either iTD (vascular invasion, lymphatic invasion, perineural invasion, and undefined cancer clusters) or nTD (cancer aggregates that had smooth or irregular shapes without an iTD component) (Fig. 1). ECLNI was defined as invasion of cancer cells into the capsular collagen tissues or into the adipose tissues beyond the capsular collagen (Fig. 1) [14]. The presence and definitions of iTDs, nTDs, and ECLNI were determined by a pathologist (S.S.) who was blinded to the patients’ clinical data.

### Statistical analyses

Clinicopathological differences between patients with and without iTDs, nTDs, or ECLNI were assessed using Fisher’s exact test, the *χ*^2^ test, or Student’s *t* test, as appropriate. Survival was analyzed using the Kaplan–Meier method, and stratified according to the various clinicopathological features. Significant differences between the survival curves were verified by the Log-rank test. Multivariate survival analyses were performed using Cox proportional hazards regression. All analyses were performed using Jump software (version 11; SAS Japan Corp., Tokyo, Japan).

## Results

### TD type (iTD vs. nTD) and clinicopathological features

First, we evaluated the effects of iTD/nTD status on clinicopathological features. Overall, iTDs and nTDs were present in 16 and 12 % of patients, respectively. Among LN-positive patients, the corresponding rates were 31 and 22 %, respectively, whereas among LN-negative patients, the corresponding rates were only 2–3 %. Significant differences were observed between iTD(+) and iTD(−) patients in terms of several clinicopathological features, including pT, pN, LN ratio, lymphatic invasion, venous invasion, nTD, ECLNI, and adjuvant chemotherapy, as well as recurrence and cancer-specific death (Table 1). Considering patients who developed recurrence, there was a higher proportion of M1b cases than M1a cases among iTD(+) patients, as compared with iTD(−) patients. No differences were observed between nTD(+) and nTD(−) patients with regard to most clinicopathological features, except for tumor site, M status, and cancer-specific death (Table 1).

**Table 1:** Associations between iTD, nTD, and ECLNI status and various clinicopathological features

	iTD			nTD			ECLNI		
Features	(+) (*n* = 60)	(−) (*n* = 325)	*P*-value	(+) (*n* = 45)	(−) (*n* = 340)	*P*-value	(+) (*n* = 60)	(−) (*n* = 119)	*P*-value
Age (years)									
Mean	67.1	67.7	0.67	65.6	67.9	0.19	68.3	66.2	0.22
Range	26–89	33–91		26–89	33–91		48–89	26–88	
≥ 70	27	150	0.89	18	159	0.43	25	54	0.75
< 70	33	175		27	181		35	65	
Gender			0.26			0.75			0.75
Male	39	183		25	197		34	64	
Female	21	142		20	143		26	55	
CEA (ng/ml)			0.56			0.75			0.50
>5	22	127		20	129		26	44	
≤5	34	164		24	174		29	64	
Tumor site			0.20			***0.0028***			***0.036***
Colon	41	248		25	264		49	78	
Rectum	19	77		20	76		11	41	
Tumor size (cm)			0.82			1.0			1.0
<5	27	144		20	151		29	57	
≥5	33	181		25	189		31	62	
Histology			1.0			0.26			1.0
Well/moderate	55	296		39	312		54	106	
Others	5	29		6	28		6	13	
pT			***0.0050***			***0.027***			***0.02***
3	43	284		33	294		41	100	
4	17	41		12	46		19	19	
LNs (n)			0.65			0.17			0.49
≥12	40	226		27	239		45	82	
<12	20	99		18	101		15	37	
pN			***<0.0001***			***<0.0001***			***<0.0001***
0	5	201		6	200		–	–	
1	28	97		21	104		30	95	
2	27	27		18	36		30	24	
LN ratio			***<0.0001***			***<0.0001***			***0.0027***
≥0.29	26	17		17	26		23	20	
<0.29	34	308		28	314		37	99	
Lymphatic invasion			***0.0015***			***0.039***			0.49
(+)	53	230		39	244		49	99	
(−)	5	87		5	87		10	14	
Venous invasion			***0.026***			***0.0069***			0.27
(+)	55	263		43	275		56	100	
(−)	3	53		1	55		3	13	
Stage			***<0.0001***			***<0.0001***			
II	5	201		6	200				
III	55	124		39	140		60	119	
Chemotherapy			***0.003***			***0.0008***			***0.033***
(+)	42	159		34	167		50	81	
(−)	18	166		11	173		10	38	
Recurrence			***<0.0001***			***<0.0001***			***0.032***
(−)	29	273		23	279		32	87	
(+) Ma	3	23		8	18		8	9	1.0
(+) Mb	28	29	***0.0012***	14	43	0.60	20	23	
Cancer-specific death			***0.0001***			0.26			1.0
(+)	10	10		4	15		5	9	
(−)	50	315		41	325		55	110	
nTD			***<0.0001***						0.34
(+)	21	24		–	–		16	23	
(−)	39	301		–	–		44	96	
ECLNI			***<0.0001***			***0.0003***			
(+)	25	35		16	44		–	–	
(−)	35	290		29	296		–	–	
iTD						***<0.0001***			***0.027***
(+)	–	–		21	39		25	32	
(−)	–	–		24	301		35	87	

*iTD* invasive-type tumor deposits, *nTD* nodular-type tumor deposits, *ECLNI* extracapsular lymph node involvement, *CEA* carcinoembryonic antigen, *LN* lymph node

**Bold text** indicates statistically significant *P*-values (<0.05)

**Fig. 2:**
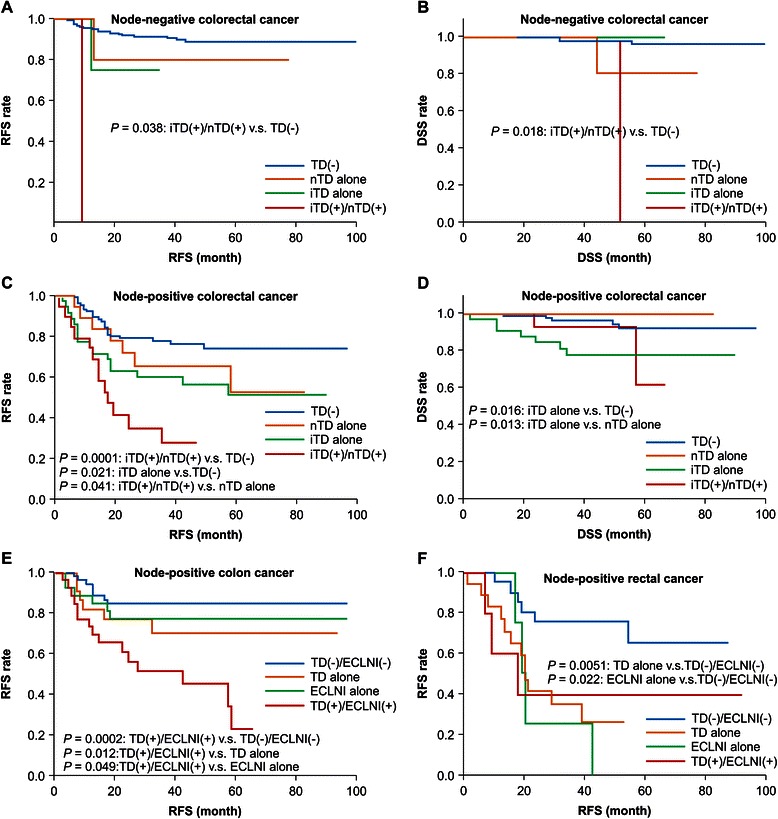
Kaplan–Meier survival curves for patients depending on TD status and ECLNI status. We analyzed associations between survival and the presence of invasive-type tumor deposits (iTDs), nodular-type tumor deposits (nTDs), and extracapsular lymph node involvement (ECLNI). These analyses were further stratified according to N status. **a** Among node-negative patients, relapse-free survival (RFS) differed significantly between the both iTD and nTD (iTD(+)/nTD(+)) and no TD (TD(−)) groups (*P* = 0.038). **b** Among node-negative patients, disease-specific survival (DSS) differed significantly between the iTD(+)/nTD(+) and TD(−) groups (*P* = 0.018). **c** Among node-positive patients, the RFS rates of the iTD(+)/nTD(+) group differed significantly from those of the TD(−) group and the nTD alone group (*P* = 0.0001 and 0.041, respectively). Among node-positive patients, RFS differed significantly between the iTD alone and TD(−) in groups (*P* = 0.021). **d** Among node-positive patients, the DSS rates of the iTD alone group differed significantly from those of the TD(−) group and the nTD alone group (*P* = 0.016 and 0.013, respectively). **e** Among node-positive patients with colon cancer, the RFS rates of the TD(+)/ECLNI(+) group differed significantly from those of the TD(−)/ECLNI(−), TD alone, and ECLNI alone groups (*P* = 0.002, 0.012, and 0.049, respectively). **f** Among node-positive patients with rectal cancer, the RFS rates of the TD(−)/ECLNI(−) group differed significantly from those of the TD alone group and the ECLNI alone group (*P* = 0.0051 and 0.022, respectively)

To clarify the prognostic differences between iTD and nTD, we assessed RFS and DSS according to both TD status and node positivity. TD status was classified into the following categories: iTD alone, nTD alone, both iTD and nTD (iTD(+)/nTD(+)), and no TD (TD(−)). On comparing iTD(+)/nTD(+) and TD(−) groups of node-negative patients, we observed significant differences in both the RFS and DSS rates (*P* = 0.038 and 0.018, respectively, Fig. 2a and b). Among node-positive patients, the RFS rates differed significantly between iTD(+)/nTD(+) and nTD alone groups, between iTD(+)/nTD(+) and TD(−) groups, and between iTD alone and TD(−) groups (Fig. 2c). Further, among node-positive patients, the DSS rates differed significantly between iTD alone group and both TD(−) group and nTD alone group (Fig. 2d). These findings indicated the importance of TD classification for the evaluation of prognosis.

### ECLNI status and clinicopathological features

The incidence of ECLNI was 34 % in patients with T3–4, N1–2 disease. The presence of ECLNI was significantly related to numerous clinicopathological features, including tumor site, pT, pN, LN ratio, iTD, adjuvant chemotherapy, and recurrence. However, the presence of ECLNI was not related to lymphatic invasion, venous invasion, or cancer-specific death (Table 1). Moreover, ECLNI status was significantly related to iTD, but not to nTD (Table 1).

### Comparison of TD and ECLNI in terms of RFS

To compare the associations of TD and ECLNI with prognosis, we investigated RFS according to TD status, ECLNI status, and cancer type (colon vs. rectum) in patients with LN metastasis. TD status and ECLNI status were classified as follows: TD alone, ECLNI alone, neither TD nor ECLNI (TD(−)/ECLNI(−)), and both TD and ECLNI (TD(+)/ECLNI(+)). Among patients with colon cancer, the RFS rates of TD(+)/ECLNI(+) group differed significantly from those of TD alone, ECLNI alone, and TD(−)/ECLNI(−) groups (Fig. 2e). Further, among patients with rectal cancer, the RFS rates of TD(−)/ECLNI(−) group differed significantly from those of TD alone and ECLNI alone groups.

These results indicated that TD and ECLNI had different prognostic associations for colon cancer and rectal cancer. The presence of both TD and ECLNI was associated with poorer RFS in colon cancer. On the other hand, presence of either TD or ECLNI was associated with poorer RFS in rectal cancer.

### Univariate and multivariate analyses of factors associated with RFS and DSS

To evaluate the prognostic significance of iTD, nTD, and ECLNI for RFS and DSS, univariate analyses were performed (Log-rank test) for a total of 15 clinicopathological factors, including factors that have been assessed by previous reports (Table 2). Four factors (pN, LN ratio, iTD, and nTD) were found to be significantly related to RFS in both colon and rectal cancer. Five factors (pT, total LN count, lymphatic invasion, venous invasion, and ECLNI) were found to be significantly related to RFS in either colon or rectal cancer. Three factors (pT, pN, and iTD) were significantly associated with both DSS and RFS in colon cancer. Finally, 2 factors (LN ratio and iTD) were significantly associated with both DSS and RFS in rectal cancer.

**Table 2:** Univariate and multivariate analyses of factors associated with RFS and DSS

	Colon				Rectum			
Factor	RFS		DSS		RFS		DSS	
	Univariate	Multivariate	Univariate	Multivariate	Univariate	Multivariate	Univariate	Multivariate
	*P*		*P*		*P*		*P*	
Age (years): ≥70	0.75	–	0.59	–	0.23	–	**0.045**	0.70
Gender: male	0.47	–	0.68	–	0.35	–	0.22	–
CEA (ng/ml): >5	0.50	–	0.69	–	0.24	–	0.34	–
pT: 3 vs. 4	**0.0029**	0.66	**<0.0001**	**0.0026**	0.33	–	0.14	–
Tumor size (cm): <5	0.49	–	0.46	–	0.054	0.47	0.75	–
Histology: well/moderate	0.71	–	0.49	–	0.51	–	0.48	–
pN: (+)	**0.0005**	0.84	**0.021**	0.47	**<0.0001**	0.079	0.22	–
Total LNs count: <12	0.41	–	0.95	–	**0.03**	0.062	0.23	–
LN ratio: >0.29	**<0.0001**	0.73	0.40		**<0.0001**	0.65	**0.026**	0.67
Lymphatic invasion: (+)	0.23	–	0.51	–	**0.045**	0.36	0.38	–
Venous invasion: (+)	**0.034**	0.14	0.13	–	0.18	–	0.24	–
Chemotherapy: (−)	0.13	–	**0.031**	0.35	0.53	–	0.39	–
iTD: (+)	**<0.0001**	0.052	**0.0038**	0.32	**<0.0001**	**0.0039**	**<0.0001**	**0.021**
nTD: (+)	**<0.0001**	0.075	0.26	–	**0.017**	**0.047**	0.65	–
ECLNI: (+)	**0.0013**	**0.02**	0.22	–	0.093	**0.025**	0.31	–

*RFS* relapse-free survival, *DSS* disease-specific survival, *CEA* carcinoembryonic antigen, *LN* lymph node, *iTD* invasive-type tumor deposits, *nTD* nodular-type tumor deposits, *ECLNI* extracapsular lymph node invasion

**Bold text** indicates statistically significant *P*-values (<0.05)

Multivariate analyses were performed, including factors that had been found to have associations with *P* < 0.1 in univariate analysis. The multivariate analyses revealed that ECLNI was significantly and independently associated with RFS in colon and rectal cancer, while iTD and nTD were significantly and independently associated with RFS in rectal cancer alone. Further, the multivariate analyses showed that pT was significantly and independently associated with DSS in colon cancer, while iTD was significantly and independently associated with DSS in rectal cancer (Table 2).

### Univariate and multivariate analyses of factors associated with the site of recurrence

Additional univariate and multivariate analyses were performed to identify factors associated with the sites of recurrence (Table 3). Using univariate analyses, we investigated the associations between 15 clinicopathological factors and liver metastasis, lung metastasis, and distant LN metastasis. All factors with *P* < 0.1 were further included in multivariate analyses. Of iTD, nTD, and ECLNI status, iTD was found to be an independent factor for liver and distant LN metastasis in colon cancer and nTD was found to be an independent factor for liver metastasis in colon cancer. We identified no independent factor for lung metastasis in colon and rectal cancer.

**Table 3:** Univariate and multivariate analyses of factors associated with the sites of recurrence

	Colon						Rectum					
	Liver		Lung		Distant LN		Liver		Lung		Distant LN	
	*P*		*P*		*P*		*P*		*P*		*P*	
Factor	Uni	Multi	Uni	Multi	Uni	Multi	Uni	Multi	Uni	Multi	Uni	Multi
Age (years): ≥70	0.55	–	0.20	–	0.054	0.15	0.057	–	0.74	–	0.64	–
Gender: Male	0.27	–	0.10	–	***0.025***	***0.041***	0.41	–	0.94	–	0.70	–
CEA (ng/ml): >5	0.14	–	0.26	–	0.54	–	0.78	–	0.59	–	0.75	–
pT: 4	***0.016***	0.88	0.51	–	***0.0097***	0.76	0.65	–	0.93	–	0.092	0.32
Tumor size (cm): <5	0.88	–	0.58	–	0.60	–	0.099	–	***0.02***	0.14	0.11	–
Histology: well/moderate	0.46	–	0.29	–	0.72	–	0.63	–	0.75	–	0.51	–
pN: (+)	***0.0003***	0.70	0.057	0.51	***0.0006***	0.64	**0.023**	0.22	***0.0003***	0.078	***0.0097***	0.55
Total LNs count: <12	0.41	–	0.17	–	0.43	–	0.099	–	0.064	0.16	0.36	–
LN ratio: ≥0.29	***0.0013***	0.99	0.098	0.49	***0.0002***	0.92	0.076	–	***<0.0001***	0.20	***0.0041***	0.44
Lymphatic invasion: (+)	***0.033***	0.37	0.50	–	0.28	–	0.13	–	***0.023***	0.48	***0.015***	0.13
Venous invasion: (+)	***0.029***	0.37	0.090	0.18	0.065	0.49	0.84	–	0.28	–	0.066	0,28
Chemotherapy: (−)	0.11	–	0.46	–	***0.0003***	0.18	0.82	–	0.30	–	0.083	0.29
iTD: (+)	***<0.0001***	***0.013***	0.80	–	**<0.0001**	**0.0055**	***0.011***	***0.0032***	***0.0008***	0.27	***0.029***	0.59
nTD: (+)	***<0.0001***	***0.010***	0.50	–	***0.0026***	0.15	0.70	–	0.12	–	0.94	–
ECLNI: (+)	***0.0004***	0.22	***0.04***	0.27	***<0.0001***	0.078	***0.027***	0.20	***0.03***	0.45	***0.022***	0.16

*RFS* relapse-free survival, *DSS* disease-specific survival, *CI* confidence interval, *CEA* carcinoembryonic antigen, *LN* lymph node, *iTD* invasive-type tumor deposits, *nTD* nodular-type tumor deposits, *ECLNI* extracapsular lymph node involvement. *Uni* univariate analysis, *Mult*i multivariate analysis

**Bold text** indicates statistically significant *P*-values (<0.05). Underlined text indicates factors that were statistically significant in multivariate analysis

## Discussion and conclusions

Here, we have evaluated the usefulness of cancer lesions (other than typical LN metastases in soft tissues) for assessing the prognosis of patients with CRC. These cancer lesions included TDs, which were further classified into iTDs and nTDs, and ECLNI (Fig. 1). We found that these factors were significantly prognostic for patients with T3–4, N0–2, M0 disease.

The presence of TDs has previously been reported as a significant prognostic factor in CRC patients receiving curative resection, and TD status has been adopted as part of both the current (7^th^ edition) and previous (5^th^ and 6^th^ editions) TNM staging systems [9–11]. However, the definition of TDs remains controversial, and it has changed in every revision of the TNM staging system since its first inclusion [2, 3, 9–11]. Rock et al. further reported that the diagnosis of TDs varied among pathologists, indicating the need for a distinct definition of TDs [13]. TDs have been reported to contain many types of cancer cells that differ in size, shape, and origin. Although classifications of TDs have been developed, the value of these different classifications has not yet been established, largely because the classification of TDs is difficult [5–8]. Here, we simply classified TDs into two types: iTD, which was defined by vascular/lymphatic/perineural invasion, and nTD, which refers to other TDs. To the best of our knowledge, our report is the first to i) show the prognostic significance of classified TDs in specimens that were resected to assess LN metastasis and ii) include a multivariate analysis adjusted for several factors, including TDs and ECLNI.

ECLNI has not yet been adopted in the TNM staging system, even though its prognostic significance has been reported in several gastrointestinal malignancies, including esophageal cancer, gastric cancer, and CRC [17]. The definition of ECLNI appears to be clearer than that of TD, although the prevalence of ECLNI varies across the available reports [5, 14–16]. Of note, TD and ECLNI status have rarely been assessed together, even though it seems necessary to distinguish between ECLNI and TDs near LN metastases [5, 15] (Fig. 1). Puppa et al. demonstrated that TD status—but not ECLNI status—was significantly associated with disease-free survival (DFS) and overall survival in their multivariate analyses [5]. Although Puppa et al. assessed DFS and overall survival, we assessed RFS and DSS in order to exclude second malignancies and deaths unrelated to cancer. On the other hand, Wind et al. found that ECLNI status—but not TD status—was a significant predictor of DFS in stage III colon cancer [15]. These different findings seem to result from the different pathological diagnoses of TD and ECLNI. Unlike these previous studies, the present study includes adjustments for several prognostic factors, such as preoperative carcinoembryonic antigen, pT, tumor site (colon or rectum), and TD types (iTD and nTD). Our results demonstrated that TD types and ECLNI were more potent prognostic factors than node positivity or LN ratio >0.29, which have been considered to be some of the strongest predictors of survival. In a multivariate analysis that excluded iTD, nTD, and ECLNI status, we found that pN (+) was a significant and independent factor for RFS rates of patients with colon (*P* = 0.034) and rectal (*P* = 0.0011) cancers. Therefore, node positivity seemed less powerful than iTD, nTD, or ECLNI as a predictor of survival in this study. Further, our results appear compatible with those of previous reports, both in the sense that vascular invasion, lymphatic invasion, and perineural invasion of cancer cells seemed to be the origins of TDs [5, 12,], and in terms of the poor prognostic outcomes that were associated with TDs [6, 7].

It is likely that the presence of iTD in soft tissues indicates the movement of cancer cells from the primary site to distant metastatic sites through the vascular, lymphatic, and/or perineural routes. In this sense, assessments of iTD status appear to be more useful than assessments of vascular invasion, lymphatic invasion, or perineural invasion in the primary tumor, which have previously been recognized as negative prognostic factors [18]. It is likely that the origins of nTD and ECLNI are also vascular invasion, lymphatic invasion, or perineural invasion of cancer cells, because these factors are associated with RFS.

In the present study, metastasis to the liver was frequent among iTD(+) patients with colon or rectal cancer and nTD(+) patients with colon cancer, whereas metastasis to the lung was not. Metastasis to the distant LNs was also frequent in iTD(+) patients with colon cancer. This finding indicates that cancer cells recognized as iTDs likely metastasize to the liver through the portal vein system, and subsequently metastasize to the distant LNs from liver in colon cancer, but not in rectal cancer. In contrast, lung metastasis is established by systems that differ from those used by iTD or nTD. The high frequency of metastasis to the distant LNs in patients with TDs is similar to the findings of a previous report [4].

There were some limitations to our work. Most importantly, this is a single-center study and pathological diagnosis was performed by a single pathologist. Multi-center studies should be performed to confirm our findings, and objective tools are indispensable for the definition and diagnosis of TDs. Thus, identifying molecular markers associated with the presence of TDs and ECLNI will aid in understanding the mechanisms of cancer metastasis.
